# Efficacy of Hypoxia-Inducible Factor Prolyl-Hydroxylase Inhibitors in Renal Anemia: Enhancing Erythropoiesis and Long-Term Outcomes in Patients with Chronic Kidney Disease

**DOI:** 10.3390/biomedicines12122926

**Published:** 2024-12-23

**Authors:** Yukina Yoshida, Tomoaki Takata, Sosuke Taniguchi, Kana Kageyama, Yudai Fujino, Hinako Hanada, Yukari Mae, Takuji Iyama, Katsuya Hikita, Hajime Isomoto

**Affiliations:** 1 Division of Gastroenterology and Nephrology, Faculty of Medicine, Tottori University, Nishi-cho 36-1, Yonago 683-8504, Tottori, Japan; 2 Kidney Center, Tottori University Hospital, Nishi-cho 36-1, Yonago 683-8504, Tottori, Japan

**Keywords:** renal anemia, HIF-PHI, RSW, ESA hyporesponsiveness, CKD

## Abstract

Background/Objectives: Renal anemia is one of the major complications associated with chronic kidney disease (CKD). Erythropoietin-stimulating agents (ESAs) are commonly used; however, some patients exhibit resistance. Hypoxia-inducible factor prolyl-hydroxylase inhibitors (HIF-PHIs) have emerged as a novel treatment for renal anemia, enhancing erythropoiesis and iron metabolism. Methods: We retrospectively analyzed laboratory data related to erythropoiesis from 105 patients with CKD before and after treatment with HIF-PHI or ESA. The dialysis initiation and mortality rates were also assessed over a median follow-up of 614 days. Results: HIF-PHI and ESA significantly increased the hemoglobin levels within 6 months of treatment (9.5 ± 1.0 to 10.7 ± 1.1, *p* < 0.01, and 9.9 ± 1.5 to 10.7 ± 1.2 g/dL, *p* < 0.01, respectively). The HIF-PHI group demonstrated a significant decrease in red cell distribution width (14.5 ± 1.9% to 13.8 ± 1.4%, *p* < 0.01), suggesting improved erythropoiesis, and exhibited a lower cumulative incidence of outcomes. The aged-adjusted multivariate analysis confirmed the independent association between HIF-PHI treatment and reduced risk of cumulative outcome (*p* = 0.042). Conclusions: HIF-PHIs can serve as an alternative to ESA for managing renal anemia in CKD, improving both hematological parameters and long-term outcomes.

## 1. Introduction

The prevalence of chronic kidney disease (CKD) is increasing [1,2], making it a major risk factor for end-stage renal disease (ESRD) and cardiovascular diseases [3,4]. Patients with CKD frequently present with multiple comorbidities, including hypertension, anemia, sarcopenia, and malnutrition [5,6,7]. In addition to the use of reno-protective agents such as renin–angiotensin–aldosterone system inhibitors and sodium–glucose cotransporter-2 inhibitors [8,9], managing renal anemia is essential for patients with CKD. Renal anemia is strongly associated with poor outcomes in patients with CKD or ESRD [10,11,12]. Until recently, erythropoietin-stimulating agents (ESAs) have been the primary treatment for renal anemia. However, some patients are resistant to ESAs, requiring higher doses and increasing their risk of mortality or cardiovascular diseases [13]. Among various conditions, deficiencies in nutrients required for hematopoiesis, such as iron, zinc, vitamins, and copper, can be easily treated. However, infection and inflammation can also lead to ESA hyporesponsiveness by disrupting iron utilization [14]. Achieving the recommended hemoglobin (Hb) levels in patients with ESA hyporesponsiveness due to inflammation remains a significant challenge.

Hypoxia-inducible factor prolyl-hydroxylase inhibitors (HIF-PHIs) represent a new class of drugs for the treatment of renal anemia. HIF is a transcription factor that stimulates the production of endogenous erythropoietin (EPO). Under normoxic conditions, prolyl-hydroxylase (PHD) binds to HIF, leading to proteasomal degradation. In response to hypoxia, PHD activity is suppressed, allowing HIF to translocate to the nucleus [15]. HIF-PHIs accelerate the HIF pathway, resulting in endogenous EPO production. Additionally, HIF-PHIs improve dysregulated iron metabolism or functional iron deficiency [16]. Functional iron deficiency occurs when the total body iron stores are adequate, but iron release from macrophages is impaired [17]. Iron availability is regulated by hepcidin, which is secreted by hepatocytes. Hepcidin secretion increases in response to inflammation, reducing iron recycling from macrophages and intestinal iron absorption [18]. As patients with CKD are predisposed to inflammation, functional iron deficiency mediated by hepcidin is a major cause of ESA-hyporesponsive anemia in CKD. HIF-PHI is a promising alternative for patients with renal anemia who are resistant to ESA [19]; however, its feasibility in the real-world clinical setting still needs to be validated.

In a clinical setting, iron status is typically assessed based on transferrin saturation (TSAT) and ferritin levels. It can also be assessed based on mean corpuscular Hb (MCH) level and red cell distribution width (RDW) [20,21]. Among them, RDW, which reflects the degree of red blood cell (RBC) heterogeneity, also indicates systemic inflammation and nutrition status [22]. High RDW indicates anisocytosis and is associated with decreased RBC deformability. RDW is also affected by inflammation and dysregulated iron metabolism. Recent studies have revealed that high RDW and vascular calcification were associated with cardiovascular events in patients with ESRD [23]. Although RDW may serve as a simple marker for dysregulated erythropoiesis in patients with CKD, its relevance and the effects of HIF-PHI and ESA on RDW still need to be fully elucidated. This study aimed to investigate the feasibility of HIF-PHI in a real-world clinical setting and explore the changes in RDW in response to HIF-PHI and ESA.

## 2. Materials and Methods

### 2.1. Study Population

This retrospective observational study included 150 patients initially treated with HIF-PHI or ESA at the Tottori University Hospital Kidney Center between January 2022 to December 2022. Patients who underwent maintenance hemodialysis or peritoneal dialysis and those with malignant tumors and hematological disorders were excluded. The appropriate therapeutic agents were determined by the attending physician, and the dosage was adjusted to achieve the target Hb levels recommended by the Japanese Society of Nephrology and the Japanese Society for Dialysis Therapy guidelines, which range from 11 to 13 g/dL [24]. We determined that a total number of 89 participants would provide the study with 95% power (*p* = 0.05; effect size 0.15). The number of participants was calculated as previously described using G*power software (version 3.1.9.6; Germany) [25,26].

### 2.2. Clinical and Laboratory Findings

The patient’s baseline characteristics and laboratory findings including RBC, Hb, hematocrit (Hct), RDW, mean corpuscular volume (MCV), mean cell Hb (MCH), mean corpuscular Hb concentration (MCHC), TSAT, ferritin, and estimated glomerular filtration rate (eGFR) levels [27] were acquired from electric medical record retrospectively. The laboratory data before and within 1, 3, and 6 months of treatment were collected. The patients were followed up until April 2024, and the outcome was defined as the initiation of dialysis or all-cause death.

### 2.3. Statistical Analysis

Continuous variables were expressed as the mean ± standard deviation. The differences between groups were analyzed using Welch’s test and a chi-square test. A two-way analysis of variance with a post hoc Dunnett’s test was used to compare the changes in laboratory data at each time point. The long-term prognosis was compared using a Kaplan–Meier survival curve analysis and a log-rank test. A Cox proportional hazards regression analysis was performed to determine the hazard ratio. A two-tailed *p* value of less than 0.05 was considered significant. StatFlex ver 7 (Artec, Osaka, Japan) and GraphPad Prism 10 (GraphPad Software, San Diego, CA, USA) were used for statistical analyses.

## 3. Results

### 3.1. Patient Characteristics

**Figure 1:**
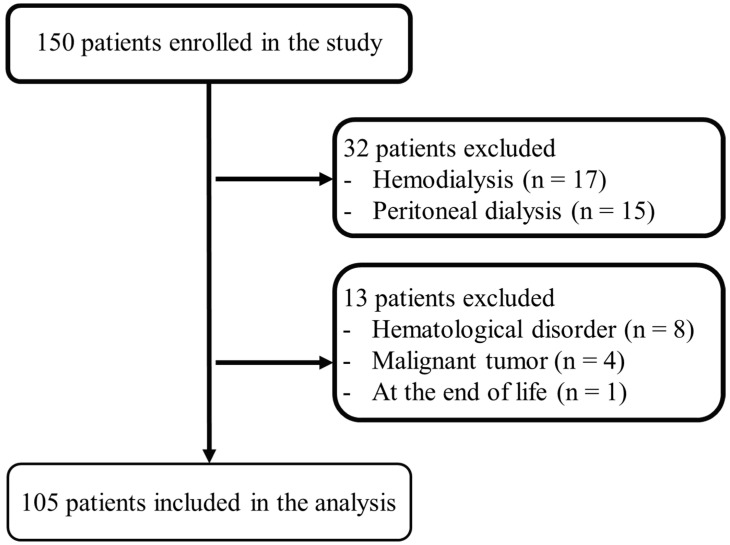
Study design. Of the 150 patients treated with HIF-PHI or ESA, 105 were included in the analysis. HIF-PHI: hypoxia-inducible factor prolyl-hydroxylase inhibitor; ESA: erythropoietin-stimulating agent.

Among 150 patients initially enrolled, 17 on maintenance hemodialysis, 15 on peritoneal dialysis, 8 with hematologic disorders, 4 with a malignant tumor, and 1 at the end of life were excluded. Hence, 105 patients were included in the final analysis (Figure 1). Patients treated with HIF-PHI showed significantly higher MCV (99.7 ± 8.2 vs. 95.6 ± 5.8 fL, *p* = 0.029) compared with those treated with ESA. Meanwhile, no significant differences were observed between the groups in the other parameters, including age (76.0 ± 10.4 vs. 72.8 ± 12.0 years, *p* = 0.22), RBC levels (3.06 ± 0.38 vs. 3.21 ± 0.51 × 10^12^/L, *p* = 0.13), Hb levels (9.5 ± 1.0 vs. 9.9 ± 1.5 g/dL, *p* = 0.14), Hct count (0.298 ± 0.034 vs. 0.306 ± 0.047 L/L, *p* = 0.40), RDW (14.5 ± 1.9 vs. 14.0 ± 1.7%, *p* = 0.26), MCH levels (31.7 ± 2.3 vs. 30.9 ± 1.9 pg, *p* = 0.16), MCHC (31.8 ± 1.5 vs. 32.4 ± 1.2 g/dL, *p* = 0.13), TSAT (29.6 ± 12.7 vs. 28.5 ± 13.5%, *p* = 0.73), ferritin levels (179.3 ± 120.4 vs. 223.1 ± 194.5 ng/dL, *p* = 0.24), C-reactive protein (CRP) (0.31 ± 0.51 vs. 0.89 ± 1.96 mg/dL, *p* = 0.071), and eGFR (24.0 ± 9.6 vs. 19.9 ± 9.4 mL/min/1.73 m^2^, *p* = 0.09) (Table 1).

### 3.2. Changes in Erythropoiesis After HIF-PHI and ESA Treatment

**Figure 2:**
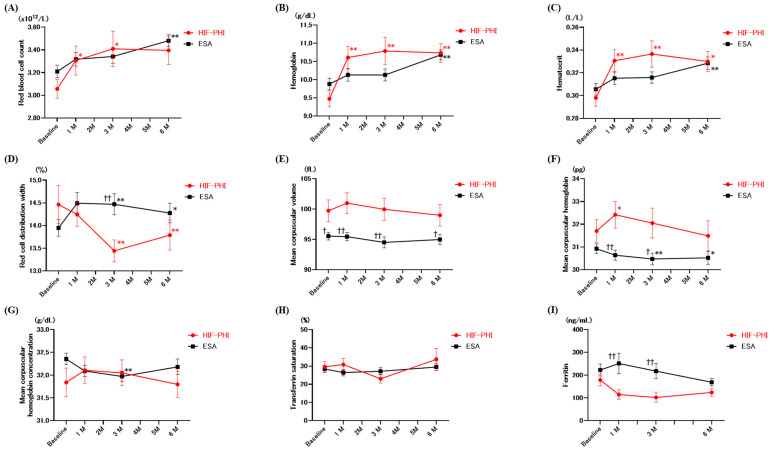
Changes in erythropoiesis after HIF-PHI and ESA treatment. Red blood cell count (**A**), hemoglobin (**B**), hematocrit (**C**), red cell distribution width (**D**), mean corpuscular volume (**E**), mean corpuscular hemoglobin (**F**), mean corpuscular hemoglobin concentration (**G**), transferrin saturation (**H**), and ferritin (**I**) were measured before and 1 month, 3 months, and 6 months after the initiation of HIF-PHI or ESA treatment. The black line represents the results from patients treated with ESA. The red line represents the results from patients treated with HIF-PHI. The bars indicate the average ± SEM. The results of Dunnett’s test compared with baseline values: * *p* < 0.05; ** *p* < 0.01. Welch’s *t*-test between the HIF-PHI and ESA groups: † *p* < 0.05; †† *p* < 0.01. HIF-PHI: hypoxia-inducible factor prolyl-hydroxylase inhibitor; ESA: erythropoietin-stimulating agent.

The changes in erythropoiesis parameters are summarized in Figure 2. During the 6-month follow-up, both HIF-PHI and ESA significantly increased the Hb levels (9.5 ± 1.0 to 10.7 ± 1.1, *p* < 0.01, and 9.9 ± 1.5 to 10.7 ± 1.2 g/dL, *p* < 0.01, respectively) and Hct count (0.298 ± 0.034 to 0.330 ± 0.038 L/L, *p* < 0.05, and 0.306 ± 0.047 to 0.328 ± 0.046 L/L, *p* < 0.01, respectively). The RBC count significantly increased 1 and 3 months after HIF-PHI treatment (3.06 ± 0.38 to 3.31 ± 0.57, *p* < 0.05, and 3.41 ± 0.68 × 10^12^/L, *p* < 0.05, respectively), and 6 months after ESA treatment (3.21 ± 0.51 to 3.48 ± 0.48 × 10^12^/L, *p* < 0.01). Interestingly, the RDW significantly decreased 6 months after HIF-PHI treatment (14.5 ± 1.9% to 13.8 ± 1.4%, *p* < 0.01), while it significantly increased in the ESA group (14.0 ± 1.7% to 14.3 ± 1.8%, *p* < 0.05). The changes in RDW (ΔRDW) after 6 months were significantly lower in the HIF-PHI group compared to those in the ESA group (−0.71 ± 2.22% vs. 0.54 ± 1.72%, *p* = 0.028). Because inflammation may affect the treatment responsiveness, we further assessed ΔRDW in patients without inflammatory condition (i.e., CRP below 1.0 mg/dL). As a result, RDW significantly reduced in the HIF-PHI group than in the ESA group (−0.74 ± 2.28% vs. 0.51 ± 1.66%, *p* = 0.036).

### 3.3. Long-Term Prognosis After HIF-PHI and ESA Treatment

**Figure 3:**
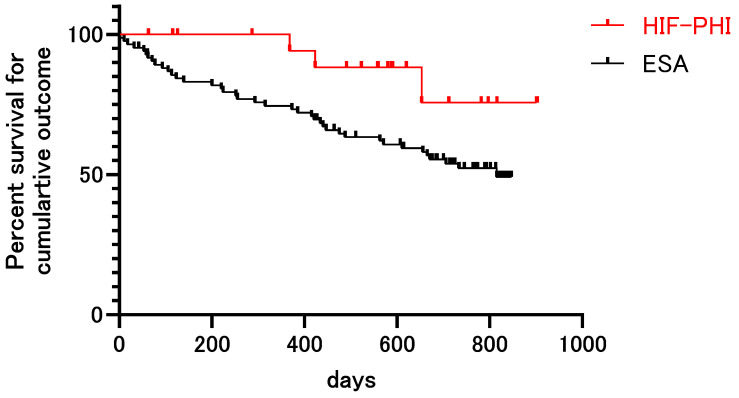
Kaplan–Meier survival analysis of long-term outcomes. Kaplan–Meier survival analysis of patients treated with HIF-PHI or ESA during a median follow-up of 614 days. The outcome was defined as the initiation of dialysis or all-cause death. The overall survival rate was significantly higher in patients treated with HIF-PHI. HIF-PHI: hypoxia-inducible factor prolyl-hydroxylase inhibitor; ESA: erythropoietin-stimulating agent.

We further investigated the long-term outcomes. Within a median follow-up of 614 days (interquartile range: 310–792), 29 patients started dialysis, while 16 patients died. The Kaplan–Meier curve analysis revealed that patients treated with HIF-PHI showed a lower incidence of cumulative outcomes during the follow-up period (*p* = 0.041) (Figure 3). Because inflammation may impact the efficacy of HIF-PHI and ESA, we analyzed the long-term prognosis in patients without inflammatory conditions. The Kaplan–Meier curve analysis in patients with CPR less than 1.0 mg/dL revealed that the HIF-PHI group showed a lower incidence of cumulative outcomes compared to that of the ESA group (*p* = 0.026). Next, we performed a multivariate Cox proportional hazards regression analysis to eliminate the impact of confounding factors on the long-term prognosis. The age-adjusted multivariate Cox proportional hazards regression analysis revealed that HIF-PHI was independently associated with a low risk for long-term renal failure and mortality (hazard ratio: 0.294; 95% confidence interval: 0.091–0.955; *p* = 0.042). To assess the impact of inflammation and renal function, CRP and the CKD stages were further added as explanatory variables. As a result, the multivariate Cox proportional hazards regression analysis adjusted for age, CRP, and the CKD stages revealed that HIF-PHI was associated with a low risk for long-term renal failure and mortality (hazard ratio: 0.857; 95% confidence interval: 0.023–0.666; *p* = 0.015).

## 4. Discussion

In the present study, we demonstrated that both HIF-PHI and ESA effectively induced hematopoiesis in patients with CKD. The HIF-PHI group exhibited immediate changes in Hb and Hct levels. Notably, the RDW significantly decreased in the HIF-PHI group but increased in the ESA group. Significant differences were also observed in the MCH and ferritin levels between the groups immediately after the treatment. These findings suggest that HIF-PHI treatment might enhance iron availability. Furthermore, patients treated with HIF-PHI had a lower incidence of dialysis initiation and mortality during the follow-up period.

We demonstrated that HIF-PHI treatment effectively improves erythropoiesis, as evidenced by the significant increases in RBC, Hb, and Hct levels, similar to those observed in ESA treatment. However, HIF-PHI treatment induced distinct changes in RDW, MCH, and ferritin levels compared with ESA treatment. Notably, the RDW significantly decreased in the HIF-PHI group but increased in the ESA group. This finding indicates a more homogenous RBC population in patients treated with HIF-PHI, potentially indicating an effective or stable erythropoiesis process. As MCH also reflects iron metabolism [21], the observed increase in MCH and decrease in ferritin levels align with the improved erythropoiesis after HIF-PHI treatment. In CKD patients, functional iron deficiency is one of the major concerns due to the elevated hepcidin levels driven by inflammation, which limits iron availability despite adequate body stores. By modulating the HIF pathway, HIF-PHI treatment may lower hepcidin levels, thereby increasing iron availability and improving erythropoiesis [28]. Although we could not assess the actual change in hepcidin levels after ESA and HIF-PHI, a larger reduction in hepcidin levels after HIF-PHI has been reported [29,30]. Stabilized HIF stimulates erythropoietin and its downstream erythrocyte, erythroferrone. Erythroferrone is reported to suppress hepcidin production from the liver, resulting in improved iron metabolism [31,32]. The effect of HIF-PHI on iron metabolism through the regulation of hepcidin is reflected by improvement in RDW [33]. These are in line with our findings that RDW changed significantly after treatment.

RDW has been used to differentiate various types of anemia, such as iron deficiency anemia, hemolytic anemia, anemia caused by ineffective erythropoiesis, and anemia caused by shortened RBC lifespan [34]. An increase in RDW indicates impaired erythrocyte production or increased erythrocyte destruction, both of which are common in CKD patients. Although the underlying mechanisms linking elevated RDW to poor outcomes are not fully understood, some investigators suggested the potential involvement of nutritional status, inflammation, and endothelial dysfunction [35,36]. Recent studies have identified RDW as a prognostic marker of cardiovascular risk in patients on hemodialysis and peritoneal dialysis [37,38]. Similarly, RDW has been identified as a feasible prognostic marker in patients with CKD. It was found to be an independent predictor of in-hospital mortality in patients with CKD [39]. Another cohort study in Japan demonstrated that patients with CKD who had an RDW over 13.6% experienced a higher incidence of ESRD and mortality during a median follow-up of 4.7 years [40]. Other observational studies also reported RDW as a predictive marker of cardiovascular, cerebrovascular, and microvascular outcome [41,42,43,44].

We also observed that the long-term outcomes were more favorable in the HIF-PHI group, with a lower incidence of dialysis initiation and mortality. Considering the effective erythropoiesis observed in the HIF-PHI group, as indicated by the reduction in RDW, this finding underscores the potential of HIF-PHI as a therapeutic option not only for managing renal anemia but also for improving overall patient prognosis [40]. Notably, these favorable outcomes could be observed after adjustment for inflammation and the CKD stages. Because improvements in hematopoiesis and iron metabolism might contribute to a more favorable prognosis after HIF-PHI treatment [45], further study is required to uncover its protective benefits beyond erythropoiesis.

Despite these promising findings, this study has several limitations. Due to the small sample size, retrospective design, and lack of randomization, the findings should be interpreted with caution. A larger, randomized controlled trial is needed to confirm these results and establish HIF-PHIs as a standard treatment for ESA-resistant renal anemia. Additionally, further investigation into the mechanisms by which HIF-PHIs influence iron metabolism and erythropoiesis is warranted. Finally, the safety profile of HIF-PHIs and their long-term outcomes need more attention.

## 5. Conclusions

In conclusion, this study provided novel insights into the effectiveness of HIF-PHI and ESA in managing renal anemia in patients with CKD. The findings support the potential of HIF-PHI as a viable option due to its favorable effects on RDW, iron metabolism, and long-term outcomes.

## Figures and Tables

**Table 1:** Patient’s baseline characteristics.

		HIF-PHI	ESA	*p* Value
Number		21	84	
Age (year)		76.0 ± 10.4	72.8 ± 12.0	0.22
Sex	(male/female)	10/11	49/35	0.22
Agents				
	Daprodustat/Vadadustat/Roxadustat	10/6/5		
	CERA/Darbepoetin alfa		59/25	
Laboratory data at baseline				
	Red blood cell (×10^12^/L)	3.06 ± 0.38	3.21 ± 0.51	0.13
	Hemoglobin (g/dL)	9.5 ± 1.0	9.9 ± 1.5	0.14
	Hematocrit (L/L)	0.298 ± 0.034	0.306 ± 0.047	0.40
	RDW (%)	14.5 ± 1.9	14.0 ± 1.7	0.26
	Mean corpuscular volume (fL)	99.7 ± 8.2	95.6 ± 5.8	0.029
	Mean cell hemoglobin (pg)	31.7 ± 2.3	30.9 ± 1.9	0.16
	Mean corpuscular hemoglobin concentration (g/dL)	31.8 ± 1.5	32.4 ± 1.2	0.13
	Transferrin saturation (%)	29.6 ± 12.7	28.5 ± 13.5	0.73
	Ferritin (ng/dL)	179.3 ± 120.4	223.1 ± 194.5	0.24
	C reactive protein (mg/dL)	0.31 ± 0.51	0.89 ± 1.96	0.071
	eGFR (mL/min/1.73 m^2^)	24.0 ± 9.6	19.9 ± 9.4	0.09
CKD stager (n, %)				0.15
	3	5 (23.8)	12 (14.3)	
	4	13 (61.9)	42 (50.0)	
	5	3 (14.3)	30 (35.7)	

CERA, continuous erythropoietin receptor activator; RDW, red blood cell distribution width; eGFR, estimated glomerular filtration rate.

## Data Availability

The datasets used and/or analyzed during the current study are available from the corresponding author on reasonable request.
